# Differences in Out-of-Pocket Expenditures for Healthcare Services Among Medicare Beneficiaries by Demographics

**DOI:** 10.3390/healthcare14152331

**Published:** 2026-08-01

**Authors:** Boon Peng Ng, Nicha Thiamwong, Jacqueline LaManna, Georgianne Tiu Hawkins, Yingru Li, Chanhyun Park

**Affiliations:** 1 College of Nursing, University of Central Florida, Orlando, FL 32827, USA; 2 Disability, Aging, and Technology Cluster, University of Central Florida, Orlando, FL 32816, USA; 3 College of Medicine, University of Central Florida, Orlando, FL 32827, USA; 4 Department of Health Management and Policy, University of Kentucky, Lexington, KY 40536, USA; 5 Department of Sociology, University of Central Florida, Orlando, FL 32816, USA; 6 College of Pharmacy, The University of Texas at Austin, Austin, TX 78712, USA

**Keywords:** Medicare, out-of-pocket expenditures, disparities, demographics

## Abstract

**Highlights:**

**What are the main findings?**

- Average per capita out-of-pocket expenditures for healthcare services among Medicare beneficiaries were $2511 in 2019 and $2641 in 2021, with notable variation across demographic groups.
- Non-Hispanic Black beneficiaries—overall and among those aged 65–74—consistently spent less than their non-Hispanic White counterparts despite having lower incomes and greater comorbidities.

**What are the implications of the main findings?**

- Out-of-pocket expenditures for healthcare services remained burdensome, with persistent demographic disparities.
- Underlying drivers require further study, and policies/practices are needed to reduce financial strain on Medicare beneficiaries.

**Abstract:**

**Background/Objectives**: Many Medicare beneficiaries often face substantial financial strain due to healthcare costs. This study examined out-of-pocket expenditures for healthcare services by demographics among Medicare beneficiaries in 2019 and 2021. **Methods**: A repeated cross-sectional study analyzed the 2019 and 2021 Medicare Current Beneficiary Survey Cost Files of beneficiaries aged ≥65 years (2019, n = 6895; 2021, n = 6094). Survey-weighted generalized linear models with a log link and gamma distribution with a three-way interaction of sex (male, female), age (65–74, ≥75), and race/ethnicity (non-Hispanic White, non-Hispanic Black, Hispanic, and Other), adjusted for covariates, and to 2021 USD, were conducted to estimate per capita annual out-of-pocket expenditures for healthcare services, excluding premiums. **Results**: Estimated average per capita annual out-of-pocket expenditures for healthcare services were $2511 in 2019 and $2641 in 2021. Differences in out-of-pocket expenditures were observed among various demographic groups in 2019 and 2021. Non-Hispanic Blacks had lower out-of-pocket expenditures than non-Hispanic Whites in both years (in 2021, $2139 [1761–2517] vs. $2730 [2580–2879]). Beneficiaries aged 65–74 years who were non-Hispanic Black had lower out-of-pocket expenditures than non-Hispanic Whites in the same age group for both years. A significant proportion of non-Hispanic Blacks had lower incomes (in 2021, income < $25,000, 46.1% vs. 17.0%) and more comorbidities (in 2021, comorbidities ≥ 4, 55.6% vs. 44.7%), compared to non-Hispanic Whites in both years. Similar differences in incomes and comorbidities were observed among those aged 65–74 years of both groups. **Conclusions**: Medicare beneficiaries face substantial out-of-pocket burdens for healthcare services, with persistent demographic disparities. Understanding contributing factors and implementing effective policies/practices are essential to reduce/mitigate high out-of-pocket expenditures for beneficiaries.

## 1. Introduction

Although 94% of Americans aged ≥65 years are covered by Medicare [1], they spend almost twice as much of their total household expenses on healthcare compared to the general population [1]. These expenses are associated with Medicare’s cost-sharing requirement, including deductibles and copayments [2], and are especially burdensome for many because they have limited financial resources. Approximately 50% of these individuals have annual incomes below $29,650 [3], and primarily rely on Social Security benefits and limited savings [3]. This limited financial capacity can severely restrict their ability to access and pay for necessary medical care [3]. About one in four Americans aged ≥65 years has reduced spending on essentials, including food, medication, or utilities, due to healthcare costs [1].

During the COVID-19 pandemic, Medicare waived some out-of-pocket expenses, including deductibles and copayments, to help older Americans access COVID-19 testing and treatment. However, not all hospitalization expenses were fully covered. On average, Medicare Advantage patients spent $1536 out-of-pocket per COVID-19-related hospitalization, suggesting that waivers for cost-sharing did not cover all associated costs [4].

Previous studies on out-of-pocket expenditures among Medicare beneficiaries have primarily focused on chronic conditions and sociodemographic factors, mainly with a single demographic group based upon sex, age, or race/ethnicity [4,5,6,7,8]. Information about out-of-pocket expenditures among Medicare beneficiaries during the pandemic is limited. Even less is known about differences among demographic subgroups categorized by sex, age, and race/ethnicity. For example, compare the out-of-pocket expenditures of non-Hispanic Black female beneficiaries aged 65–74 with those of non-Hispanic White female beneficiaries of the same age. Additionally, out-of-pocket expenditures among certain demographic subgroups are important to examine, as they may reflect underlying disparities in healthcare access, use, and affordability. Lower out-of-pocket expenditures may not signal reduced financial burden; instead, they may indicate barriers to care or unmet healthcare needs [9,10].

Thus, this study aimed to estimate out-of-pocket healthcare expenditures among different demographic subgroups characterized by sex, age, and race/ethnicity in 2019 and in 2021. This information can provide a better understanding of the financial challenges these populations face and inform the development of targeted interventions to alleviate the financial burden on underserved populations.

## 2. Materials and Methods

### 2.1. Data and Study Population

This repeated cross-sectional secondary data study used the 2019 and 2021 Medicare Current Beneficiary Survey (MCBS) Cost Supplement Public Use Files (PUFs). The data contain information on the utilization and costs of healthcare services used by Medicare beneficiaries, based on the MCBS and administrative records. They include copayments, deductibles, and services not covered by Medicare; however, they exclude premiums [11]. These publicly available secondary datasets include only community-dwelling beneficiaries [11]. More information about the data can be found on the CMS website [11]. The IRB had determined that the proposed activity did not constitute human subject research.

The study population included 6895 and 6094 Medicare beneficiaries aged ≥65 years in 2019 and in 2021, respectively.

### 2.2. Measures

Dependent variable

The total annual per capita out-of-pocket payments for eight healthcare services (i.e., dental, home health, hearing, inpatient hospital, medical provider, outpatient hospital, prescribed medicine, and vision) were used. For 2019, the out-of-pocket expenditures were converted to 2021 U.S. dollars using the Consumer Price Index for all urban consumers [12].

Independent variables

We included a three-way interaction of three demographic categorical variables of sex (male or female), age (65–74 or ≥75), and race/ethnicity (non-Hispanic White, non-Hispanic Black, Hispanic, or Other). All the demographic variables were categorized based on the predefined groupings provided in the MCBS Cost Supplement PUFs.

Covariates

All estimated out-of-pocket expenditures were adjusted using ten socioeconomic, comorbidity, and utilization variables available in the dataset: household income (<$25,000, ≥$25,000), number of chronic conditions (0–1, 2–3, ≥4), inpatient health care utilization visits (0, 1, ≥2), outpatient encounters (0, 1–3, 4–6, ≥7), prescribed medications (0, 1–10, 11–20, 21–30, ≥31), medical provider visits (0, 1–5, 6–10, 11–15, ≥16), home health (0, ≥1), dental (0, 1–2, ≥3), hearing (0, ≥1), and vision events (0, 1, ≥2). These covariates were included to account for socioeconomic status, disease burden, and healthcare utilization patterns that may influence out-of-pocket spending estimates.

### 2.3. Statistical Analysis

Descriptive statistics were conducted to examine the characteristics of study participants. Unadjusted out-of-pocket expenditures with average and 95% confidence intervals by demographics were calculated. Two generalized linear models with a log link function and gamma distribution (to account for the positive skew of out-of-pocket expenditures) with a three-way interaction of sex, age, and race/ethnicity, adjusted for covariates, were conducted to estimate annual per capita out-of-pocket expenditures in 2019 and in 2021. Average annual out-of-pocket expenses were estimated by using the average marginal effects method from the models. All analyses used survey weights to account for the complex survey design of MCBS. Analyses were conducted using SAS Enterprise Guide 8.3 (SAS Institute Inc., Cary, NC, USA) and Stata/SE 17 (StataCorp LLC, College Station, TX, USA).

## 3. Results

Table 1 displays the sociodemographic characteristics and healthcare utilization of the study population. In 2019, 45.1% of Medicare beneficiaries were male, and 60.6% were aged 65–74 years. Of study beneficiaries, 76.0% were non-Hispanic White, while 9.0% were non-Hispanic Black, 8.1% were Hispanic, and 6.9% identified as Other. Among beneficiaries, 73.7% had incomes ≥ $25,000, and 47.1% had 2–3 chronic conditions. Approximately 89.7% of beneficiaries had no inpatient events, and 8.0% had 1 inpatient event. About one-fourth of beneficiaries had no outpatient events, while 37.9% had 1–3 outpatient events. The Supplemental Materials provide additional detailed descriptions of the sociodemographic characteristics and healthcare utilization of the demographic subgroups. The demographic characteristics in 2021 were similar to those in 2019. However, the number of chronic conditions and some healthcare utilization rates varied (Table 1).

Table 2 shows the unadjusted average per capita annual out-of-pocket expenditures for Medicare beneficiaries by sex, age, and race/ethnicity in 2021 USD. In 2019, per capita annual out-of-pocket expenditures for Medicare beneficiaries were $2351. The out-of-pocket expenditures showed no meaningful differences by sex ($2364 vs. $2340). Beneficiaries aged ≥75 years had $2640 in out-of-pocket expenditures, while those aged 65–74 years spent $2163. Non-Hispanic Whites had the highest out-of-pocket expenditures among racial/ethnic groups, compared with non-Hispanic Blacks, who had the lowest expenditures ($2613 vs. $1400). The Supplemental Materials provide additional detailed descriptions of the unadjusted average per capita annual out-of-pocket expenditures for the demographic subgroups.

In 2021, the unadjusted average per capita annual out-of-pocket spending for Medicare beneficiaries was $2519 (Table 2). Male beneficiaries had slightly higher spending than female beneficiaries ($2556 vs. $2490). Beneficiaries aged ≥75 years spent more than beneficiaries aged 65–74 years ($2937 vs. $2245). Non-Hispanic White beneficiaries had the highest out-of-pocket expenditures, while Hispanic beneficiaries had the lowest ($2761 vs. $1431). The Supplemental Materials provide additional detailed descriptions of the unadjusted average per capita annual out-of-pocket expenditures for the demographic subgroups.

Table 3 provides the estimated annual per capita out-of-pocket expenditures for Medicare beneficiaries in 2021 USD, derived from generalized linear models using a log link and gamma distribution. In 2019, the estimated average per capita annual out-of-pocket expenditure was $2511. There were no significant differences in out-of-pocket expenditures by sex or age group. Non-Hispanic Whites had significantly higher out-of-pocket expenditures than non-Hispanic Blacks ($2618, 95% CI [$2449–$2786] vs. $1982 [$1623–$2342]), and significantly higher spending than beneficiaries identified as Other. For sex-age groups, out-of-pocket expenditures did not differ significantly between male beneficiaries aged 65–74 years and female beneficiaries aged 75 years. For sex-race/ethnicity groups, male non-Hispanic Whites spent significantly more than female beneficiaries identified as Other ($2617, 95% CI [$2382–$2851] vs. $1764 [$1431–$2098]); however, out-of-pocket expenditures for other groups were not statistically different. For age-race/ethnicity groups, beneficiaries aged 65–74 years who were non-Hispanic White spent significantly more than those aged 65–74 years who were non-Hispanic Black ($2569, 95% CI [$2326–$2813] vs. $1874 [$1441–$2306]). They also spent more than beneficiaries aged 65–74 years who were identified as Other. For sex-age-race/ethnicity groups, male beneficiaries aged 65–74 years who were non-Hispanic White had significantly higher expenditures than male beneficiaries aged 65–74 years who were identified as Other ($2554, 95% CI [$2236–$2871] vs. $1753, 95% CI [$1428–$2079]).

In 2021, the estimated average per capita annual out-of-pocket expenditure was $2641 (Table 3). Non-Hispanic Whites had significantly higher out-of-pocket expenditures than non-Hispanic Blacks ($2730, 95% CI [$2580–$2879] vs. $2139 [$1761–$2517]). For sex-age groups, differences in spending between female beneficiaries aged 65–74 years and male beneficiaries aged ≥75 years were not significant. For sex-race/ethnicity groups, male non-Hispanic White beneficiaries spent significantly more than male Hispanic beneficiaries ($2796, 95% CI [$2598–$2994] vs. $1758 [$1285–$2230]) and more than female non-Hispanic Black beneficiaries. For age-race/ethnicity groups, beneficiaries aged 65–74 years who were non-Hispanic White spent significantly more than those aged 65–74 years who were non-Hispanic Black ($2699, 95% CI [$2481–$2917] vs. $1740 [$1396–$2085]) and more than those aged 65–74 years who were Hispanic. For sex-age-race/ethnicity groups, male beneficiaries aged 65–74 years who were non-Hispanic White had significantly higher spending than male beneficiaries aged 65–74 years who were Hispanic ($2659, 95% CI [$2390–$2928] vs. $1454, 95% CI [$980–$1928]), and also spent more than female beneficiaries aged 65–74 years who were non-Hispanic Black or Other.

The Supplemental Tables S1–S6 present socioeconomic characteristics, comorbidities, and Medicare coverage benefits stratified by sex, age, and race/ethnicity to provide context for the findings using MCBS Cost Supplement PUF and MCBS PUF for both years.

## 4. Discussion

Despite the availability of healthcare coverage, many Medicare beneficiaries continued to face a significant out-of-pocket burden, even during the pandemic when various waivers for COVID-19-related treatment costs were in place. We found that Medicare beneficiaries spent over $2500 annually on out-of-pocket healthcare services in both years. Differences in out-of-pocket expenditures were observed across demographic subgroups in 2019 and in 2021. Multiple factors, such as access to needed care and enrolling in the dual eligible program, likely contributed to these differences in out-of-pocket spending. Given the complexities of healthcare financing, providers play a critical role in helping their patients, which can be both challenging and important. Providers need specific training and evidence-based strategies to screen for and address patients’ financial stress [13]. Many older adults may postpone or forgo nonurgent medical care due to difficulty paying bills, which can lead to worsening health conditions and higher overall healthcare costs.

Our findings showed that the out-of-pocket expenditures varied by race/ethnicity, with a significant difference between non-Hispanic Black and non-Hispanic White beneficiaries. This is consistent with past research conducted by the Kaiser Family Foundation (KFF) and may be attributed to various factors [14]. We hypothesized (based on Supplemental Tables S1–S6) that these differences likely occur in part due to greater eligibility and higher enrollment rates of many non-Hispanic Black beneficiaries in both the dual eligible program (those who enroll in the dual eligible program receive full Medicaid benefits or help with premiums and/or cost-sharing through the Medicare Savings Programs) [15,16] and Medicare Part D low-income subsidy program (also known as Extra Help) [17] compared with non-Hispanic White beneficiaries. In 2019, a higher proportion of non-Hispanic Black beneficiaries were enrolled in the dual-eligible program (28.8% vs. 5.8%) and low-income subsidy program (36.0% vs. 7.4%) than non-Hispanic White beneficiaries (Tables S3 and S4). The dual-eligible program likely plays a significant role in helping non-Hispanic Black beneficiaries with their out-of-pocket spending. For example, Medicare Savings Programs (MSPs) help eligible beneficiaries pay Medicare premiums and/or cost sharing expenses [15]. Enrollment in MSPs has been associated with annual out-of-pocket healthcare savings of up to $1144 [18]. Among these programs, Qualified Medicare Beneficiary (QMB) enrollees are less likely to delay necessary care due to cost and less likely to encounter difficulty paying medical bills than beneficiaries who were eligible but not enrolled, indicating a reduced financial burden [19]. The low-income subsidy (LIS) program helps cover beneficiaries’ Medicare drug plan monthly premiums, annual deductibles, and copayments. Eligible beneficiaries who do not participate in the LIS program incur approximately $518 more in annual out-of-pocket costs than LIS enrollees, fill fewer prescriptions, and are more likely to forgo medications due to cost [20]. Unfortunately, many beneficiaries may be eligible for these programs but not enrolled [18,21], resulting in difficulties paying their healthcare expenses [22]. Therefore, providers and advocates should encourage older adults to check their eligibility for these programs by contacting their local Area Agency on Aging, State Health Insurance Assistance Program, or benefits enrollment center [21].

Additionally, some non-Hispanic Black beneficiaries with lower incomes (Table S6) may be eligible for dual-eligible program/low-income subsidy programs but not enrolled. Also, individuals whose incomes are too high (or not low enough) to meet eligibility criteria for these programs (fall into the coverage gap) may be unable to afford out-of-pocket spending, and therefore bear the greatest financial burden [14]. According to the 2022 Commonwealth Fund Biennial Health Insurance Survey, about one in five Medicare beneficiaries aged ≥65 years were underinsured (high out-of-pocket costs relative to their income) and struggled to pay their premiums [2]. It was reported that racial/ethnic minority beneficiaries (i.e., Black beneficiaries) were more likely to be underinsured or less likely to be enrolled in supplemental insurance coverage (e.g., Medigap that can help to cover beneficiaries’ cost-sharing) [23]. Therefore, efforts to screen patients who may be under financial stress and refer them to programs such as HUD Housing Assistance, SNAP Food Benefits, and Property Tax Relief should be considered [24].

Our findings also showed that beneficiaries aged 65–74 years who were non-Hispanic Black had lower out-of-pocket expenditures than non-Hispanic Whites in the same age group. This is also likely in part due to higher enrollment in dual-eligible and low-income subsidy programs among this demographic. Tables S3 and S4 show that in 2019, 26.4% (vs. 6.0%) of beneficiaries aged 65–74 years who were non-Hispanic Black enrolled in a dual-eligible program and 34.1% (vs. 7.7%) enrolled in a low-income subsidy program, significantly higher percentages than their White counterparts. However, it is important to note that many non-Hispanic Black beneficiaries experienced higher chronic disease burdens (Table S2). According to KFF, minority Medicare beneficiaries are more likely to report poor health and have higher rates of chronic conditions, such as hypertension and diabetes [25]. They are also less likely to visit a doctor yet more likely to be admitted to the hospital or visit the emergency room than White peers [25]. Additionally, although relatively few Medicare beneficiaries report problems accessing care, a larger proportion of Black beneficiaries than White beneficiaries report trouble obtaining the care they need [25]. Therefore, the lower out-of-pocket spending we observed needs to be discussed in the context of many factors such as access to quality of care, comorbidities, quality of life, and other social determinants of health to fully understand the issue.

Interestingly, no significant differences were observed between age groups (65–74 vs. ≥75 years), which may partially reflect the use of predefined age categories in CMS data that limit more granular comparisons. In contrast, a KFF report using more refined age stratification found significantly higher estimates among individuals aged ≥85 years compared with those aged 65–74 years [14]. This is likely because individuals aged ≥85 years have a higher prevalence of chronic conditions, greater need for long-term care services, and higher rates of hospitalization, therefore higher out-of-pocket spending [26].

Finally, we observed differences in out-of-pocket spending among different demographic groups before (2019) and during the COVID-19 pandemic (2021), although identifying the factors associated with each difference is complex and beyond the scope of this study. The findings are not surprising, as many beneficiaries’ care-seeking behaviors were altered during the pandemic. Telehealth use became more widespread, with 81.2% of beneficiaries reporting that their usual providers offered telehealth during the COVID-19 pandemic [27]. Hispanic and Non-Hispanic Black beneficiaries were more likely to report using telehealth services than non-Hispanic White beneficiaries during this period [28]. Additionally, disparities in access to healthcare services by sociodemographic factors and comorbidity burden were observed among Medicare beneficiaries during the pandemic [29]. However, the long-term impact of these altered care-seeking behaviors on Medicare beneficiaries’ out-of-pocket costs and health outcomes warrants further investigation.

### Limitations

This study has several limitations. We were unable to calculate the out-of-pocket income ratio, which would provide more detailed insight into the burden of out-of-pocket spending on different demographic groups. The data did not include premium information, only covering payments related to the eight services; therefore, the total out-of-pocket spending is likely higher than reported. Unfortunately, the MCBS Cost Supplemental PUFs did not have dual-eligible program (or Medicare Savings Programs) and low-income subsidy program related variables, preventing us from investigating the impact of these programs on out-of-pocket spending among demographic groups. Although we controlled income, comorbidities, and healthcare utilization in our regression models, other variables may also impact out-of-pocket spending. Issues with recollection and other biases inherent in survey data could affect our estimates; however, these effects are likely minimal, as survey-reported out-of-pocket costs were validated using available administrative records and billing information [11]. Because the MCBS Cost Supplement PUFs use predefined categorizations for demographic variables included in this study, different classification schemes may produce different analytic insights.

Future research should examine how disparities in out-of-pocket spending relate to subsequent health outcomes across demographic subgroups, including potential effects on medication adherence, preventive care use, morbidity, and mortality. Applying the Blinder–Oaxaca decomposition could also provide deeper insight into why groups differ in average spending by separating the spending gap into explained and unexplained components. In addition, qualitative studies exploring why individuals eligible for Medicare Savings Programs or the low-income subsidy remain unenrolled would be valuable for improving outreach strategies, ensuring successful enrollment, and understanding how program participation may influence beneficiaries’ out-of-pocket costs.

## 5. Conclusions

Significant variations in out-of-pocket expenditures were observed across several demographic groups in 2019 and 2021, particularly between non-Hispanic White and Black beneficiaries and the corresponding 65–74 age group. Although out-of-pocket expenditures are a major concern for many beneficiaries, they impact different groups unequally [30]. Cost-cutting strategies that burden White Medicare beneficiaries could lead to catastrophic hardships for minority beneficiaries [31]. As high healthcare costs disproportionately affect low-income populations, minorities, and those who are not enrolled in dual-eligible (or Medicare Savings Programs) and low-income subsidy programs. This could result in medical debt for many [32]. Delayed or forgone treatment, decreased medication adherence, and lower utilization of preventive care services [30] can all negatively impact beneficiaries’ health outcomes [33,34]. Finally, in a clinical setting, screening patients for unmet basic needs and referring them to social safety net programs should be considered and implemented via EHR systems [35].

## Figures and Tables

**Table 1:** Sociodemographic characteristics and health care utilization of the study’s Medicare beneficiaries aged ≥65 Years.

	2019		2021	
	Unweighted N	Weighted N (%)	Unweighted N	Weighted N (%)
Total	6895	48.4 million	6094	51.6 million
Sex				
Male	3092	21.8 million (45.1)	2706	23.0 million (44.7)
Female	3803	26.6 million (54.9)	3388	28.5 million (55.3)
Age group				
65–74 years	2854	29.3 million (60.6)	2651	31.1 million (60.3)
≥75 years	4041	19.1 million (39.4)	3443	20.5 million (39.7)
Sex and age group				
Male, 65–74 years	1322	13.7 million (28.3)	1216	14.3 million (27.7)
Male, ≥75 years	1770	8.1 million (16.8)	1490	8.7 million (16.9)
Female, 65–74 years	1532	15.6 million (32.3)	1435	16.8 million (32.6)
Female, ≥75 years	2271	10.9 million (22.6)	1953	11.7 million (22.8)
Race/ethnicity				
Non-Hispanic White	5216	36.8 million (76.0)	4746	40.1 million (77.8)
Non-Hispanic Black	581	4.3 million (9.0)	459	4.4 million (8.5)
Hispanic	713	3.9 million (8.1)	602	4.1 million (8.0)
Other	385	3.4 million (6.9)	287	2.9 million (5.6)
Sex and Race/ethnicity				
Male, Non-Hispanic White	2373	16.7 million (34.6)	2108	18.0 million (34.9)
Male, Non-Hispanic Black	234	1.9 million (3.9)	191	1.9 million (3.8)
Male, Hispanic	293	1.5 million (3.2)	259	1.7 million (3.2)
Male, Other	192	1.7 million (3.4)	148	1.4 million (2.8)
Female, Non-Hispanic White	2843	20.1 million (41.5)	2638	22.1 million (42.9)
Female, Non-Hispanic Black	347	2.4 million (5.0)	268	2.5 million (4.8)
Female, Hispanic	420	2.4 million (4.9)	343	2.5 million (4.8)
Female, Other	193	1.7 million (3.5)	139	1.5 million (2.8)
Age and Race/ethnicity				
65–74 years, Non-Hispanic White	2123	21.9 million (45.2)	2014	23.7 million (46.0)
65–74 years, Non-Hispanic Black	256	2.8 million (5.7)	226	2.9 million (5.5)
65–74 years, Hispanic	285	2.4 million (5.0)	287	2.7 million (5.3)
65–74 years, Other	190	2.3 million (4.7)	124	1.8 million (3.5)
≥75 years, Non-Hispanic White	3093	14.9 million (30.8)	2732	16.4 million (31.8)
≥75 years, Non-Hispanic Black	325	1.6 million (3.2)	233	1.6 million (3.0)
≥75 years, Hispanic	428	1.5 million (3.1)	315	1.4 million (2.7)
≥75 years, Other	195	1.1 million (2.2)	163	1.1 million (2.1)
Sex, age, and Race/ethnicity				
Male, 65–74 years, Non-Hispanic White	978	10.2 million (21.2)	921	10.9 million (21.2)
Male, 65–74 years, Non-Hispanic Black	117	1.3 million (2.7)	106	1.4 million (2.7)
Male, 65–74 years, Hispanic	123	1.0 million (2.0)	124	1.1 million (2.0)
Male, 65–74 years, Other	104	1.2 million (2.5)	65	0.9 million (1.8)
Female, 65–74 years, Non-Hispanic White	1145	11.6 million (24.0)	1093	12.8 million (24.8)
Female, 65–74 years, Non-Hispanic Black	139	1.5 million (3.0)	120	1.5 million (2.8)
Female, 65–74 years, Hispanic	162	1.4 million (3.0)	163	1.7 million (3.2)
Female, 65–74 years, Other	86	1.1 million (2.3)	59	0.9 million (1.7)
Male, ≥75 years, Non-Hispanic White	1395	6.5 million (13.4)	1187	7.0 million (13.7)
Male, ≥75 years, Non-Hispanic Black	117	0.6 million (1.2)	85	0.6 million (1.1)
Male, ≥75 years, Hispanic	170	0.6 million (1.2)	135	0.6 million (1.2)
Male, ≥75 years, Other	88	0.5 million (1.0)	83	0.5 million (1.0)
Female, ≥75 years, Non-Hispanic White	1698	8.4 million (17.4)	1545	9.4 million (18.1)
Female, ≥75 years, Non-Hispanic Black	208	1.0 million (2.0)	148	1.0 million (1.9)
Female, ≥75 years, Hispanic	258	0.9 million (1.9)	180	0.8 million (1.6)
Female, ≥75 years, Other	107	0.6 million (1.2)	80	0.6 million (1.1)
Income				
<$25,000	2144	12.8 million (26.3)	1553	12.1 million (23.6)
≥$25,000	4751	35.7 million (73.7)	4541	39.4 million (76.4)
Number of chronic conditions				
0–1	1938	16.1 million (33.3)	772	7.8 million (15.2)
2–3	3343	22.8 million (47.1)	2250	19.9 million (38.6)
≥4	1614	9.5 million (19.6)	3072	23.8 million (46.2)
Inpatient events				
0	6104	43.4 million (89.7)	5559	47.2 million (91.5)
1	598	3.9 million (8.0)	427	3.5 million (6.9)
≥2	193	1.1 million (2.3)	108	0.9 million (1.7)
Outpatient events				
0	1528	12.0 million (24.7)	1437	13.4 million (26.0)
1–3	2595	18.3 million (37.9)	2305	19.8 million (38.3)
4–6	1210	8.1 million (16.8)	1072	8.5 million (16.5)
≥7	1562	10.0 million (20.6)	1280	9.9 million (19.2)
Prescribed medicine events				
0	279	3.2 million (6.6)	240	3.0 million (5.9)
1–10	1107	9.3 million (19.2)	1096	10.5 million (20.4)
11–20	1468	10.3 million (21.4)	1332	11.3 million (21.9)
21–30	1325	8.7 million (18.0)	1177	9.5 million (18.5)
≥31	2716	16.9 million (34.9)	2249	17.2 million (33.4)
Medical provider events				
0	198	1.9 million (4.0)	100	1.2 million (2.3)
1–5	960	7.8 million (16.2)	811	8.3 million (16.2)
6–10	1095	7.8 million (16.2)	1009	8.7 million (16.8)
11–15	883	6.1 million (12.6)	801	6.6 million (12.7)
≥16	3759	24.7 million (51.1)	3373	26.8 million (51.9)
Home health events				
0	6178	44.5 million (91.9)	5481	47.2 million (91.6)
≥1	717	3.9 million (8.1)	613	4.4 million (8.4)
Dental events				
0	2937	19.3 million (39.9)	2371	19.9 million (38.6)
1–2	2259	16.5 million (34.1)	2085	17.9 million (34.7)
≥3	1699	12.6 million (26.0)	1638	13.8 million (26.7)
Hearing events				
0	5824	42.1 million (86.9)	5155	44.7 million (86.7)
≥1	1071	6.3 million (13.1)	939	6.8 million (13.3)
Vision events				
0	2788	20.7 million (42.7)	2335	21.1 million (41.0)
1	2087	14.7 million (30.3)	2001	17.0 million (32.9)
≥2	2020	13.0 million (26.9)	1758	13.5 million (26.1)

Note: Each year of data is presented in two columns: the first reports the unweighted number of beneficiaries, and the second reports the weighted count and corresponding percentage. These results describe the sociodemographic characteristics and health care utilization patterns of the study population. For example, in 2019, 45.1% (21.8 million) of beneficiaries were male and 54.9% (26.6 million) were female.

**Table 2:** Unadjusted average per capita annual out-of-pocket expenditures of the study’s Medicare beneficiaries aged ≥65 years by Sex, Age, and Race/Ethnicity in 2021 USD.

	2019			2021		
Variable	Average	95% CI		Average	95% CI	
Overall	2351	2228	2474	2519	2396	2643
Sex						
Male	2364	2204	2525	2556	2388	2724
Female	2340	2175	2505	2490	2328	2652
Age group						
65–74 years	2163	2008	2319	2245	2090	2400
≥75 years	2640	2477	2802	2937	2765	3109
Sex and age group						
Male, 65–74 years	2081	1868	2295	2123	1929	2317
Male, ≥75 years	2842	2627	3057	3266	2971	3560
Female, 65–74 years	2235	2030	2440	2348	2122	2575
Female, ≥75 years	2489	2254	2725	2692	2496	2888
Race/ethnicity						
Non-Hispanic White	2613	2458	2767	2761	2601	2920
Non-Hispanic Black	1400	1099	1700	1492	1237	1747
Hispanic	1479	1223	1735	1431	1105	1756
Other	1730	1460	2000	2293	1710	2876
Sex and Race/ethnicity						
Male, Non-Hispanic White	2581	2385	2777	2765	2565	2964
Male, Non-Hispanic Black	1542	1035	2050	1800	1323	2278
Male, Hispanic	1557	1158	1955	1140	859	1422
Male, Other	1870	1485	2255	2596	1808	3384
Female, Non-Hispanic White	2639	2433	2846	2757	2545	2970
Female, Non-Hispanic Black	1289	1015	1563	1246	925	1568
Female, Hispanic	1429	1072	1786	1624	1103	2145
Female, Other	1594	1247	1941	1995	1201	2788
Age and Race/ethnicity						
65–74 years, Non-Hispanic White	2406	2201	2610	2464	2258	2671
65–74 years, Non-Hispanic Black	1344	961	1728	1273	986	1561
65–74 years, Hispanic	1509	1102	1915	1312	940	1683
65–74 years, Other	1530	1219	1840	2292	1536	3047
≥75 years, Non-Hispanic White	2916	2723	3109	3189	3000	3379
≥75 years, Non-Hispanic Black	1498	1153	1842	1893	1244	2542
≥75 years, Hispanic	1432	1167	1696	1659	982	2336
≥75 years, Other	2164	1549	2779	2296	1448	3143
Sex, age, and Race/ethnicity						
Male, 65–74 years, Non-Hispanic White	2287	2018	2555	2255	2023	2486
Male, 65–74 years, Non-Hispanic Black	1470	778	2162	1578	1086	2070
Male, 65–74 years, Hispanic	1513	927	2099	841	591	1092
Male, 65–74 years, Other	1441	1102	1780	2855	1663	4047
Female, 65–74 years, Non-Hispanic White	2511	2234	2788	2644	2342	2946
Female, 65–74 years, Non-Hispanic Black	1234	935	1532	981	705	1258
Female, 65–74 years, Hispanic	1506	965	2048	1610	1019	2201
Female, 65–74 years, Other	1625	1144	2106	1712	892	2532
Male, ≥75 years, Non-Hispanic White	3047	2790	3303	3557	3222	3891
Male, ≥75 years, Non-Hispanic Black	1699	1073	2325	2356	1288	3424
Male, ≥75 years, Hispanic	1629	1121	2137	1667	1100	2235
Male, ≥75 years, Other	2977	1763	4192	2154	1470	2838
Female, ≥75 years, Non-Hispanic White	2816	2531	3101	2912	2700	3124
Female, ≥75 years, Non-Hispanic Black	1374	963	1784	1634	713	2554
Female, ≥75 years, Hispanic	1309	998	1620	1653	531	2775
Female, ≥75 years, Other	1537	998	2077	2426	943	3909

**Table 3:** Estimated annual per capita out-of-pocket expenditures for Medicare beneficiaries in 2021 USD based on generalized linear models.

	2019			2021		
Variable	Estimated Average	95% CI		Estimated Average	95% CI	
Overall	2511	2371	2650	2641	2514	2768
Sex						
Male	2534	2329	2739	2744	2566	2922
Female	2494	2319	2669	2559	2403	2715
Age group						
65–74 years	2456	2262	2649	2599	2412	2787
≥75 years	2590	2426	2753	2723	2564	2882
Sex and age group						
Male, 65–74 years	2447	2171	2722	2622	2374	2870
Male, ≥75 years	2662	2440	2884	2905	2638	3172
Female, 65–74 years	2467	2206	2727	2562	2339	2785
Female, ≥75 years	2524	2304	2743	2577	2351	2804
Race/ethnicity						
Non-Hispanic White	2618	2449	2786	2730	2580	2879
Non-Hispanic Black	1982	1623	2342	2139	1761	2517
Hispanic	2147	1741	2552	2078	1450	2707
Other	2052	1701	2403	2629	1952	3306
Sex and Race/ethnicity						
Male, Non-Hispanic White	2617	2382	2851	2796	2598	2994
Male, Non-Hispanic Black	1943	1463	2423	2719	2033	3405
Male, Hispanic	2290	1733	2846	1758	1285	2230
Male, Other	2396	1801	2990	3193	2079	4307
Female, Non-Hispanic White	2618	2408	2828	2674	2490	2858
Female, Non-Hispanic Black	2015	1577	2453	1664	1267	2061
Female, Hispanic	2027	1416	2637	2337	1256	3418
Female, Other	1764	1431	2098	2191	1355	3027
Age and Race/ethnicity						
65–74 years, Non-Hispanic White	2569	2326	2813	2699	2481	2917
65–74 years, Non-Hispanic Black	1874	1441	2306	1740	1396	2085
65–74 years, Hispanic	2162	1538	2787	1735	1390	2081
65–74 years, Other	1858	1498	2219	2761	1856	3665
≥75 years, Non-Hispanic White	2679	2508	2849	2763	2597	2929
≥75 years, Non-Hispanic Black	2120	1623	2616	2600	1887	3313
≥75 years, Hispanic	2127	1647	2606	2483	1159	3807
≥75 years, Other	2298	1579	3018	2493	1547	3439
Sex, age, and Race/ethnicity						
Male, 65–74 years, Non-Hispanic White	2554	2236	2871	2659	2390	2928
Male, 65–74 years, Non-Hispanic Black	1823	1137	2510	2017	1585	2449
Male, 65–74 years, Hispanic	2349	1485	3213	1454	980	1928
Male, 65–74 years, Other	1753	1428	2079	4060	2126	5995
Female, 65–74 years, Non-Hispanic White	2582	2258	2907	2731	2458	3004
Female, 65–74 years, Non-Hispanic Black	1916	1449	2382	1518	1049	1986
Female, 65–74 years, Hispanic	2006	1085	2927	1962	1452	2471
Female, 65–74 years, Other	1946	1388	2503	1714	1134	2294
Male, ≥75 years, Non-Hispanic White	2697	2452	2942	2957	2662	3252
Male, ≥75 years, Non-Hispanic Black	2095	1508	2683	3547	2094	5001
Male, ≥75 years, Hispanic	2215	1544	2886	2116	1354	2878
Male, ≥75 years, Other	3211	1868	4554	2171	1408	2934
Female, ≥75 years, Non-Hispanic White	2663	2429	2898	2607	2384	2829
Female, ≥75 years, Non-Hispanic Black	2140	1442	2839	1837	1200	2474
Female, ≥75 years, Hispanic	2053	1359	2747	2779	478	5081
Female, ≥75 years, Other	1534	1078	1990	2753	1265	4241

## Data Availability

The original data presented in the study are openly available in the Centers for Medicare and Medicaid Services at https://data.cms.gov/medicare-current-beneficiary-survey-mcbs/medicare-current-beneficiary-survey-survey-file (accessed on 7 April 2025).

## Supplementary Materials

The following supporting information can be downloaded at: https://www.mdpi.com/article/10.3390/healthcare14152331/s1, Table S1: Income Levels of Medicare Beneficiaries Aged ≥65 by Sex, Age, and Race/Ethnicity (weighted %) (MCBS Cost Supplement PUF 2019 and 2021); Table S2: Number of Chronic Conditions Among Medicare Beneficiaries Aged ≥65 by Sex, Age, and Race/Ethnicity (weighted %) (MCBS Cost Supplement PUF 2019 and 2021); Table S3: Dual Eligibility Status of Medicare Beneficiaries Aged ≥65 by Sex, Age, and Race/Ethnicity (weighted %) (MCBS PUF 2019 and 2021); Table S4: Low-income Subsidy Status (Extra Help Program) of Medicare Beneficiaries Aged ≥65 by Sex, Age, and Race/Ethnicity (weighted %) (MCBS PUF 2019 and 2021); Table S5: Dual Eligibility Status and Low-Income Subsidy (Extra Help Program) Among Medicare Beneficiaries Aged ≥65 by Income and Federal Poverty Level (weighted %) (MCBS PUF 2019 and 2021). Table S6: Dual Eligibility Status and Low-Income Subsidy (Extra Help Program) Among Non-Hispanic Black Medicare Beneficiaries Aged ≥65 by Income and Federal Poverty Level (weighted %) (MCBS PUF 2019 and 2021).
