# Supplementary material for: Differences in Out-of-Pocket Expenditures for Healthcare Services Among Medicare Beneficiaries by Demographics
Source: Healthcare (Basel). 2026 Aug 1;14(15):2331. doi: 10.3390/healthcare14152331 (PMC13466208; doi:10.3390/healthcare14152331)
Supplement: Supplementary file 1 [file healthcare-14-02331-s001.zip › healthcare-4325894-supplementary.pdf]

Supplemental Materials

Results- Additional detailed description of the sociodemographic characteristics, healthcare utilization, and unadjusted average per-capita annual out-of-pocket expenditures of the demographic subgroups.

Table 1 displays the sociodemographic characteristics and healthcare utilization of the study population. In 2019, among sex-age groups, female beneficiaries aged 65-74 years comprised the largest group, while male beneficiaries aged ≥ 75 years represented the smallest group. Among sex-race/ethnicity groups, 41.5% were female non-Hispanic White beneficiaries, while male Hispanics comprised 3.2% of the sample. Among age-race/ethnicity groups, 45.2% were aged 65-74 years and non-Hispanic White, whereas those aged ≥ 75 years and with an Other racial/ethnicity status comprised only 2.2% of the sample. Finally, female beneficiaries aged 65-74 years who were non-Hispanic White comprised 24.0% of the sex-age-race/ethnicity groups.

Table 2 shows the unadjusted average per capita annual out-of-pocket expenditures for Medicare beneficiaries by sex, age, and race/ethnicity in 2021 USD. In 2019, among sex-race/ethnicity groups, female non-Hispanic White beneficiaries had the highest out-of-pocket expenditures ($2,639), while female non-Hispanic Black beneficiaries had the lowest ($1,289). Among age-race/ethnicity groups, beneficiaries aged ≥75 years who were non-Hispanic White had an average out-of-pocket expenditure of $2,916, while beneficiaries aged 65-74 years who were non-Hispanic Black spent $1,344. For sex-age-race/ethnicity groups, male non-Hispanic White beneficiaries aged ≥75 years had the highest spending at $3,047. In 2021, among sex-race/ethnicity groups, male non-Hispanic White beneficiaries spent the most, and male Hispanic beneficiaries spent the least ($2,765 vs. $1,140). Among age-race/ethnicity groups, beneficiaries aged ≥75 years who were non-Hispanic White had the highest spending ($3,189), while beneficiaries aged 65-74 years who were non-Hispanic Black had the lowest ($1,273). For sex-age-race/ethnicity groups, male non-Hispanic White aged ≥ 75 years had the highest out-of-pocket spending at $3,557.

Table S1 Income Levels of Medicare Beneficiaries Aged ≥ 65 by Sex, Age, and Race/Ethnicity (weighted %) (MCBS Cost Supplement PUF 2019 and 2021)

	2019		2021	
Variable	< $25,000	≥ $25,000	< $25,000	≥ $25,000
Sex				
Male	19.3	80.7	18.2	81.8
Female	32.1	67.9	27.8	72.2
Age group				
65-74 years	23.1	76.9	21.5	78.5
≥ 75 years	31.3	68.7	26.6	73.4
Sex and age group				
Male, 65-74 years	18.1	81.9	18.6	81.4
Male, ≥ 75 years	21.3	78.7	17.7	82.3
Female, 65-74 years	27.5	72.5	24.0	76.0
Female, ≥ 75 years	38.7	61.3	33.3	66.7
Race/ethnicity				
Non-Hispanic White	19.6	80.4	17.0	83.0
Non-Hispanic Black	51.9	48.1	46.1	53.9
Hispanic	54.6	45.4	57.2	42.8
Other	34.7	65.3	31.6	68.4
Sex and Race/ethnicity				
Male, Non-Hispanic White	13.7	86.3	13.0	87.0
Male, Non-Hispanic Black	43.2	56.8	37.9	62.1
Male, Hispanic	41.9	58.1	46.9	53.1
Male, Other	27.4	72.6	23.6	76.4
Female, Non-Hispanic White	24.4	75.6	20.3	79.7
Female, Non-Hispanic Black	58.6	41.4	52.6	47.4
Female, Hispanic	62.9	37.1	64.1	35.9
Female, Other	41.7	58.3	39.4	60.6
Age and Race/ethnicity				
65-74 years, Non-Hispanic White	16.5	83.5	15.5	84.5
65-74 years, Non-Hispanic Black	46.9	53.1	38.4	61.6
65-74 years, Hispanic	48.7	51.3	51.9	48.1
65-74 years, Other	30.5	69.5	27.9	72.1
≥ 75 years, Non-Hispanic White	24.0	76.0	19.2	80.8
≥ 75 years, Non-Hispanic Black	60.7	39.3	60.3	39.7
≥ 75 years, Hispanic	64.0	36.0	67.3	32.7
≥ 75 years, Other	43.6	56.4	37.6	62.4
Sex, age, and Race/ethnicity				
Male, 65-74 years, Non-Hispanic White	12.5	87.5	13.8	86.2
Male, 65-74 years, Non-Hispanic Black	42.7	57.3	34.9	65.1
Male, 65-74 years, Hispanic	37.0	63.0	45.3	54.7
Male, 65-74 years, Other	24.0	76.0	20.6	79.4
Female, 65-74 years, Non-Hispanic White	20.0	80.0	17.1	82.9
Female, 65-74 years, Non-Hispanic Black	50.5	49.5	41.7	58.3
Female, 65-74 years, Hispanic	56.4	43.6	56.1	43.9
Female, 65-74 years, Other	37.5	62.5	35.4	64.6
Male, ≥ 75 years, Non-Hispanic White	15.6	84.4	11.9	88.1
Male, ≥ 75 years, Non-Hispanic Black	44.2	55.8	45.5	54.5

Male, ≥ 75 years, Hispanic	49.9	50.1	49.8	50.2
Male, ≥ 75 years, Other	35.9	64.1	28.9	71.1
Female, ≥ 75 years, Non-Hispanic White	30.4	69.6	24.7	75.3
Female, ≥ 75 years, Non-Hispanic Black	70.9	29.1	68.7	31.3
Female, ≥ 75 years, Hispanic	72.8	27.2	80.2	19.8
Female, ≥ 75 years, Other	49.5	50.5	45.5	54.5

Table S2 Number of Chronic Conditions Among Medicare Beneficiaries Aged ≥ 65 by Sex, Age, and Race/Ethnicity (weighted %) (MCBS Cost Supplement PUF 2019 and 2021)

	2019			2021		
Variable	0-1	2-3	4+	0-1	2-3	4+
Sex						
Male	34.7	47.9	17.5	17.4	40.0	42.6
Female	32.2	46.4	21.4	13.4	37.5	49.1
Age group						
65-74 years	39.6	44.9	15.5	18.9	41.3	39.8
≥ 75 years	23.6	50.4	26.0	9.5	34.5	56.0
Sex and age group						
Male, 65-74 years	40.0	46.3	13.7	21.4	42.7	35.9
Male, ≥ 75 years	25.7	50.4	23.9	10.8	35.5	53.7
Female, 65-74 years	39.2	43.7	17.1	16.9	40.0	43.1
Female, ≥ 75 years	22.1	50.4	27.5	8.5	33.8	57.7
Race/ethnicity						
Non-Hispanic White	34.9	47.0	18.1	15.6	39.7	44.7
Non-Hispanic Black	25.7	50.1	24.2	12.5	31.9	55.6
Hispanic	28.2	47.4	24.4	14.1	36.8	49.2
Other	31.9	43.5	24.6	14.7	36.4	48.8
Sex and Race/ethnicity						
Male, Non-Hispanic White	35.3	47.9	16.8	17.8	40.9	41.3
Male, Non-Hispanic Black	29.5	48.6	21.8	14.3	30.5	55.2
Male, Hispanic	32.5	48.9	20.6	19.2	42.0	38.8
Male, Other	35.8	47.4	16.8	13.8	39.0	47.2
Female, Non-Hispanic White	34.5	46.3	19.2	13.9	38.7	47.5
Female, Non-Hispanic Black	22.7	51.2	26.1	11.0	33.0	56.0
Female, Hispanic	25.3	47.7	27.0	10.7	33.3	56.0
Female, Other	28.2	39.7	32.1	15.6	33.9	50.5
Age and Race/ethnicity						
65-74 years, Non-Hispanic White	41.7	44.8	13.5	19.8	42.6	37.6
65-74 years, Non-Hispanic Black	31.2	47.5	21.2	15.0	33.7	51.3
65-74 years, Hispanic	31.8	45.7	22.5	17.1	39.8	43.1
65-74 years, Other	38.2	41.7	20.1	17.1	37.8	45.1
≥ 75 years, Non-Hispanic White	25.0	50.2	24.8	9.7	35.4	55.0
≥ 75 years, Non-Hispanic Black	15.8	54.7	29.5	7.8	28.7	63.5
≥ 75 years, Hispanic	22.4	50.1	27.5	8.2	31.0	60.8
≥ 75 years, Other	18.3	47.4	34.2	10.9	34.2	54.9
Sex, age, and Race/ethnicity						
Male, 65-74 years, Non-Hispanic White	41.2	46.4	12.3	22.4	44.0	33.6
Male, 65-74 years, Non-Hispanic Black	32.4	47.2	20.4	15.5	33.4	51.1
Male, 65-74 years, Hispanic	36.3	44.1	19.5	23.9	44.3	31.8
Male, 65-74 years, Other	40.9	46.2	12.9	15.4	39.7	44.9
Female, 65-74 years, Non-Hispanic White	42.1	43.4	14.5	17.5	41.4	41.0
Female, 65-74 years, Non-Hispanic Black	30.2	47.8	22.0	14.6	34.0	51.5
Female, 65-74 years, Hispanic	28.7	46.7	24.5	12.8	36.9	50.3

Female, 65-74 years, Other	35.4	36.8	27.9	18.9	35.7	45.3
Male, ≥ 75 years, Non-Hispanic White	26.1	50.2	23.7	10.8	36.1	53.1
Male, ≥ 75 years, Non-Hispanic Black	23.2	51.8	25.0	11.3	23.2	65.5
Male, ≥ 75 years, Hispanic	26.3	51.4	22.3	10.7	38.0	51.3
Male, ≥ 75 years, Other	22.6	50.6	26.8	11.2	37.8	51.0
Female, ≥ 75 years, Non-Hispanic White	24.1	50.2	25.7	8.8	34.8	56.3
Female, ≥ 75 years, Non-Hispanic Black	11.3	56.4	32.3	5.8	31.7	62.5
Female, ≥ 75 years, Hispanic	20.0	49.3	30.7	6.4	25.9	67.7
Female, ≥ 75 years, Other	15.0	45.0	39.9	10.7	31.0	58.4

Table S3 Dual Eligibility Status of Medicare Beneficiaries Aged ≥ 65 by Sex, Age, and Race/Ethnicity (weighted %) (MCBS PUF 2019 and 2021)

	2019		2021	
Variable	Nondual	Dual	Nondual	Dual
Sex				
Male	91.7	8.3	90.6	9.4
Female	86.8	13.2	85.7	14.3
Age group				
65-74 years	89.5	10.5	87.6	12.4
≥ 75 years	88.2	11.8	88.3	11.7
Sex and age group				
Male, 65-74 years	91.2	8.8	89.2	10.8
Male, ≥ 75 years	92.5	7.5	92.8	7.2
Female, 65-74 years	88.0	12.0	86.2	13.8
Female, ≥ 75 years	85.1	14.9	85.1	14.9
Race/ethnicity				
Non-Hispanic White	94.2	5.8	93.4	6.6
Non-Hispanic Black	71.2	28.8	69.5	30.5
Hispanic	64.9	35.1	59.3	40.7
Other	81.9	18.1	82.3	17.7
Sex and Race/ethnicity				
Male, Non-Hispanic White	95.7	4.3	95.0	5.0
Male, Non-Hispanic Black	75.3	24.7	75.5	24.5
Male, Hispanic	72.6	27.4	63.4	36.6
Male, Other	86.9	13.1	86.1	13.9
Female, Non-Hispanic White	93.0	7.0	92.0	8.0
Female, Non-Hispanic Black	68.3	31.7	65.1	34.9
Female, Hispanic	59.7	40.3	56.5	43.5
Female, Other	77.1	22.9	78.9	21.1
Age and Race/ethnicity				
65-74 years, Non-Hispanic White	94.0	6.0	92.6	7.4
65-74 years, Non-Hispanic Black	73.6	26.4	70.9	29.1
65-74 years, Hispanic	70.0	30.0	63.9	36.1
65-74 years, Other	86.1	13.9	85.1	14.9
≥ 75 years, Non-Hispanic White	94.6	5.4	94.5	5.5
≥ 75 years, Non-Hispanic Black	66.9	33.1	66.7	33.3
≥ 75 years, Hispanic	57.2	42.8	51.3	48.7
≥ 75 years, Other	75.1	24.9	77.4	22.6
Sex, age, and Race/ethnicity				
Male, 65-74 years, Non-Hispanic White	95.1	4.9	94.1	5.9
Male, 65-74 years, Non-Hispanic Black	74.6	25.4	72.1	27.9
Male, 65-74 years, Hispanic	75.0	25.0	65.2	34.8
Male, 65-74 years, Other	89.8	10.2	84.7	15.3
Female, 65-74 years, Non-Hispanic White	93.0	7.0	91.3	8.7
Female, 65-74 years, Non-Hispanic Black	72.8	27.2	70.0	30.0
Female, 65-74 years, Hispanic	66.4	33.6	63.0	37.0
Female, 65-74 years, Other	82.4	17.6	85.5	14.5
Male, ≥ 75 years, Non-Hispanic White	96.8	3.2	96.5	3.5
Male, ≥ 75 years, Non-Hispanic Black	77.1	22.9	84.4	15.6

Male, ≥ 75 years, Hispanic	68.7	31.3	60.4	39.6
Male, ≥ 75 years, Other	82.0	18.0	88.8	11.2
Female, ≥ 75 years, Non-Hispanic White	92.9	7.1	93.0	7.0
Female, ≥ 75 years, Non-Hispanic Black	61.6	38.4	57.1	42.9
Female, ≥ 75 years, Hispanic	50.1	49.9	45.2	54.8
Female, ≥ 75 years, Other	68.8	31.2	68.7	31.3

Table S4 Low-income Subsidy Status (Extra Help Program) of Medicare Beneficiaries Aged ≥ 65 by Sex, Age, and Race/Ethnicity (weighted %) (MCBS PUF 2019 and 2021)

	2019		2021	
Variable	No premium subsidy	Premium subsidy	No premium subsidy	Premium subsidy
Sex				
Male	89.6	10.4	89.2	10.8
Female	83.7	16.3	83.4	16.6
Age group				
65-74 years	86.6	13.4	85.8	14.2
≥ 75 years	86.0	14.0	86.3	13.7
Sex and age group				
Male, 65-74 years	88.7	11.3	87.7	12.3
Male, ≥ 75 years	91.1	8.9	91.6	8.4
Female, 65-74 years	84.7	15.3	84.1	15.9
Female, ≥ 75 years	82.3	17.7	82.4	17.6
Race/ethnicity				
Non-Hispanic White	92.6	7.4	92.0	8.0
Non-Hispanic Black	64.0	36.0	65.2	34.8
Hispanic	59.2	40.8	55.6	44.4
Other	77.6	22.4	79.8	20.2
Sex and Race/ethnicity				
Male, Non-Hispanic White	94.5	5.5	94.3	5.7
Male, Non-Hispanic Black	70.6	29.4	70.7	29.3
Male, Hispanic	66.7	33.3	59.3	40.7
Male, Other	83.2	16.8	84.7	15.3
Female, Non-Hispanic White	91.1	8.9	90.2	9.8
Female, Non-Hispanic Black	59.3	40.7	61.1	38.9
Female, Hispanic	54.2	45.8	53.1	46.9
Female, Other	72.2	27.8	75.4	24.6
Age and Race/ethnicity				
65-74 years, Non-Hispanic White	92.3	7.7	91.4	8.6
65-74 years, Non-Hispanic Black	65.9	34.1	66.7	33.3
65-74 years, Hispanic	63.6	36.4	59.8	40.2
65-74 years, Other	81.0	19.0	83.4	16.6
≥ 75 years, Non-Hispanic White	93.1	6.9	92.8	7.2
≥ 75 years, Non-Hispanic Black	60.7	39.3	62.2	37.8
≥ 75 years, Hispanic	52.5	47.5	48.3	51.7
≥ 75 years, Other	72.1	27.9	73.6	26.4
Sex, age, and Race/ethnicity				
Male, 65-74 years, Non-Hispanic White	93.5	6.5	93.2	6.8
Male, 65-74 years, Non-Hispanic Black	69.8	30.2	68.2	31.8
Male, 65-74 years, Hispanic	67.6	32.4	60.1	39.9
Male, 65-74 years, Other	85.5	14.5	83.9	16.1
Female, 65-74 years, Non-Hispanic White	91.2	8.8	89.9	10.1
Female, 65-74 years, Non-Hispanic Black	62.5	37.5	65.4	34.6
Female, 65-74 years, Hispanic	60.7	39.3	59.6	40.4
Female, 65-74 years, Other	76.5	23.5	82.9	17.1
Male, ≥ 75 years, Non-Hispanic White	96.1	3.9	95.9	4.1

Male, ≥ 75 years, Non-Hispanic Black	72.2	27.8	77.2	22.8
Male, ≥ 75 years, Hispanic	65.1	34.9	57.9	42.1
Male, ≥ 75 years, Other	79.1	20.9	86.2	13.8
Female, ≥ 75 years, Non-Hispanic White	90.9	9.1	90.5	9.5
Female, ≥ 75 years, Non-Hispanic Black	54.5	45.5	54.0	46.0
Female, ≥ 75 years, Hispanic	44.7	55.3	41.9	58.1
Female, ≥ 75 years, Other	65.6	34.4	64.0	36.0

Table S5 Dual Eligibility Status and Low-Income Subsidy (Extra Help Program) Among Medicare Beneficiaries Aged ≥ 65 by Income and Federal Poverty Level (weighted %) (MCBS PUF 2019 and 2021)

	2019		2021	
Dual Eligibility	Nondual	Dual	Nondual	Dual
Income				
<$25,000	62.0	38.0	55.3	44.7
>=$25,00	99.2	0.8	98.1	1.9
Poverty Level				
<=100% of the Federal Poverty Level	36.4	63.6	30.8	69.2
>100% and <=120% of the Federal Poverty	59.2	40.8	55.5	44.5
>120% and <=135% of the Federal Poverty	79.3	20.7	66.2	33.8
>135% and <=200% of the Federal Poverty	93.7	6.3	88.1	11.9
>200% of the Federal Poverty Level	99.6	0.4	98.9	1.1
Low-income Subsidy Indicator	No premium subsidy	Premium subsidy	No premium subsidy	Premium subsidy
Income				
<$25,000	54.2	45.8	48.7	51.3
>=$25,00	98.6	1.4	97.7	2.3
Poverty Level				
<=100% of the Federal Poverty Level	28.0	72.0	24.9	75.1
>100% and <=120% of the Federal Poverty	51.1	48.9	48.9	51.1
>120% and <=135% of the Federal Poverty	69.0	31.0	56.8	43.2
>135% and <=200% of the Federal Poverty	89.6	10.4	83.7	16.3
>200% of the Federal Poverty Level	99.0	1.0	98.7	1.3

Table S6 Dual Eligibility Status and Low-Income Subsidy (Extra Help Program) Among Non-Hispanic Black Medicare Beneficiaries Aged ≥ 65 by Income and Federal Poverty Level (weighted %) (MCBS PUF 2019 and 2021)

	2019		2021	
Dual Eligibility	Nondual	Dual	Nondual	Dual
Income				
<$25,000	47.4	52.6	38.4	61.6
>=$25,00	97.1	0.9	93.3	6.7
Poverty Level				
<=100% of the Federal Poverty Level	29.4	70.6	19.4	80.6
>100% and <=120% of the Federal Poverty	47.7	52.3	40.5	59.5
>120% and <=135% of the Federal Poverty	68.4	31.6	39.4	60.6
>135% and <=200% of the Federal Poverty	88.5	11.5	80.1	19.9
>200% of the Federal Poverty Level	97.9	2.1	95.5	4.5
Low-income Subsidy Indicator	No premium subsidy	Premium subsidy	No premium subsidy	Premium subsidy
Income				
<$25,000	36.2	63.8	30.9	69.1
>=$25,00	94.2	5.8	91.3	8.7
Poverty Level				
<=100% of the Federal Poverty Level	18.1	81.9	37.7	62.3
>100% and <=120% of the Federal Poverty	37.7	62.3	59.3	40.7
>120% and <=135% of the Federal Poverty	59.8	40.2	66.2	33.8
>135% and <=200% of the Federal Poverty	80.8	19.2	86.6	13.4
>200% of the Federal Poverty Level	94.6	5.4	99.2	0.8
